# Dyslipidemia and Eye Diseases in the Adult Chinese Population: The Beijing Eye Study

**DOI:** 10.1371/journal.pone.0026871

**Published:** 2012-03-26

**Authors:** Shuang Wang, Liang Xu, Jost B. Jonas, Ya Xing Wang, Qi Sheng You, Hua Yang

**Affiliations:** 1 Beijing Institute of Ophthalmology, Beijing Tongren Hospital, Capital Medical University, Beijing, China; 2 Department of Ophthalmology, Medical Faculty Mannheim of the Ruprecht-Karls-University, Heidelberg, Germany; University of Tor Vergata, Italy

## Abstract

To determine associations between dyslipidemia and ocular diseases, the population-based Beijing Eye Study 2006 examined 3251 subjects (age≥45 years) who underwent a detailed ophthalmic examination and biochemical blood analysis. Dyslipidemia was defined as any of the following: hypercholesterolemia (total cholesterol concentration≥5.72 mmol/L (220 mg/dL)) or hypertriglyceridemia (triglyceride concentration≥1.70 mmol/L (150 mg/dL)) or low high-density lipoprotein-cholesterol (HDL-C concentration≤0.91 mmol/L (35 mg/dL)). Biochemical blood examinations were available for 2945 (90.6%) subjects. After adjustment for age, gender, habitation region, body mass index, self reported income, blood glucose concentration, diastolic blood pressure and smoking, dyslipidemia was significantly associated with higher intraocular pressure (*P*<0.001) and beta zone of parapapillary atrophy (*P* = 0.03). Dyslipidemia was not significantly associated with the prevalence of glaucoma (*P* = 0.99), retinal vein occlusions (*P* = 0.92), diabetic retinopathy (*P* = 0.49), presence of retinal vascular abnormalities such as focal or general arteriolar narrowing, age-related macular degeneration (*P* = 0.27), nuclear cataract (*P* = 0.14), cortical cataract (*P* = 0.93), and subcapsular cataract (*P* = 0.67). The results make one conclude that, controlled for systemic and socioeconomic parameters, dyslipidemia was not associated with common ophthalmic disorders including glaucoma and age-related macular degeneration.

## Introduction

Dyslipidemia, a major systemic disorder, is one of the most important risk factors for cardiovascular disease which is a major cause of morbidity and a leading contributor to mortality worldwide [1]–[4]. Due to its pronounced impact on many organs of the body, dyslipidemia has also been indirectly or directly linked to a wide range of eye diseases, including age-related macular degeneration, glaucoma, retinal vein occlusions and hypertensive and diabetic retinopathy [5]–[22].

Most of these studies, however, were conducted on Western populations, in which the prevalence, risk factors, treatment strategies and therapy frequencies of dyslipidemia may be different from Asian populations. And most of these studies have often been hospital-based investigations with the potential risk of a referral bias. And most of the studies usually addressed the relationship between dyslipidemia and a single ocular parameter only (e.g., age-related macular degeneration or cataract only) without taking into account inter-relationships between various ocular diseases, or without talking account associations between dyslipidemia and other systemic factors as potentially confounding factors, such as body mass index, socioeconomic background and diabetes.

We, therefore, conducted the present study to assess in a relatively population-based study the relationships between dyslipidemia and major eye diseases such as age-related macular degeneration, glaucoma, cataract, hypertensive retinopathy and diabetic retinopathy, with taking into account associations between dyslipidemia and other systemic disorders, such as level of education, body height and body mass index. Although this was a cross-sectional approach which by definition cannot give clues on the future development of diseases in association of baseline data such as the presence of dyslipidemia, the relatively large study population of more than 3000 participants, the population-based study sample recruitment, and the simultaneous inclusion of all major ocular diseases and some of the major systemic parameters may allow to arrive at results which may be more conclusive than those which have been available in previous investigations.

## Results

The study included 2945 (90.6%) subjects (1671 women) for whom serum lipids measurements were available. The mean age was 60.4±10.0 years (median: 60 years; range: 45–89 years). Out of the 2945 individuals, 1545 (52.5%) subjects (840 women) came from the rural region, and 1400 (47.5%) subjects (831 women) came from the urban region. The subjects from the rural region compared with the subjects from the urban region were significantly younger (56.9±9.0 years versus 63.6±9.9 years; *P*<0.001), and had significantly lower monthly income (399±310 Yuan versus 2177±594 Yuan; *P*<0.001) and lower level of education (*P*<0.001). The participants of the survey 2006 compared with the non-participants were significantly younger (55.3±0.1 years versus 58.6±11.6 years; *P*<0.001), came more often from the rural region than from the urban region (1500/1751 versus 473/714; *P*<0.001), and had a higher level of education (*P* = 0.001). There were no significant differences in gender (females/males 1838/1413 versus 668/521; *P* = 0.84).

Mean levels of total cholesterol, HDL cholesterol, LDL cholesterol, and triglycerides were 4.92±1.01 mmol/L, 1.61±0.36 mmol/L, 2.88±0.85 mmol/L, and 1.76±1.29 mmol/L, respectively. A hypercholesterolemia (total cholesterol concentration ≥5.72 mmol/L (220 mg/dL)) was found in 19.0±0.7%, a hypertrigylceridemia (triglyceride concentration ≥1.70 mmol/L (150 mg/dL)) in 35.5±0.9%, and abnormally low high-density lipoprotein-cholesterol (HDL-C concentration ≤0.91 mmol/L (35 mg/dL)) in 1.7±0.2% of the study population. Dyslipidemia was found for 1332 subjects (prevalence: 45.1±0.9% (mean ± standard error; 95%CI: 43.3%, 46.9%). A positive history for dyslipidemia was described by 842 (28.5%) subjects, so that the total prevalence of dyslipidemia was 1654/2951 or 56.1±0.9% (95%CI: 54.3%, 57.8%). For all further statistical analysis, the total prevalence of dyslipidemia (abnormal blood examinations results and / or positive history for dyslipidemia) was taken.

With respect to non-ocular parameters, the presence of dyslipidemia was significantly associated with increasing age (*P*<0.001), female gender (*P*<0.001), urban region (*P* = 0.001), body mass index (*P*<0.001), income (*P* = 0.01), blood glucose concentration (*P*<0.001), diastolic blood pressure (*P* = 0.02), and smoking (*P* = 0.04).

With respect to ophthalmic parameters, the prevalence of dyslipidemia was associated significantly (bivariate analysis) with increasing intraocular pressure (*P*<0.001), larger optic disc area (*P*<0.001), larger area of beta zone of parapapillary atrophy (*P* = 0.001), and increased presence of cortical cataract (*P* = 0.02) (Tables 1,2). There were no significant associations between the prevalence of dyslipidemia and the prevalence of retinal vein occlusions (*P* = 0.21), diabetic retinopathy (*P* = 0.12), open-angle glaucoma (*P* = 0.99), angle closure glaucoma (*P* = 0.60), and age-related macular degeneration (*P* = 0.08 for early type; *P* = 0.30 for late type), refractive error (*P* = 0.91), best corrected visual acuity (*P* = 0.07), area of alpha zone of parapapillary atrophy (*P* = 0.39), generalized retinal arteriolar narrowing (*P* = 0.64), focal arteriolar narrowing (*P* = 0.69), arterio-venous nicking (*P* = 0.23), and the degree of nuclear cataract (*P* = 0.08) and subcapsular cataract (*P* = 0.66) (Tables 1,2).

**Table 1:** Differences in Ocular Parameters in Subjects with and without Dyslipidemia in the Beijing Eye Study 2006 (Categorical variables).

	No Dyslipidemia		Dyslipidemia		Odds Ratio	95% CI	*P*- value
	n/N	%	n/N	%			
Focal arteriolar narrowing	136/1503	9.0%	106/1230	8.6%	0.95	0.73, 1.23	0.69
Arterio-venous nicking	128/1504	8.5%	121/1230	9.8%	1.17	0.90, 1.52	0.23
Generalized narrowing	295/1515	19.5%	251/1214	20.2%	1.05	0.87, 1.26	0.64
Diabetic retinopathy	57/1593	3.6%	62/1310	4.7%	1.34	0.93, 1.93	0.12
Nuclear cataract	113/1604	7.0%	117/1327	8.8%	1.28	0.98, 1.67	0.08
Post. subcapsular cataract	57/1503	3.8%	52/1263	4.1%	1.09	0.74, 1.59	0.66
Cortical cataract	102/1503	6.8%	115/1263	9.1%	1.38	1.04, 1.81	0.02
Retinal vein occlusion	21/1604	1.3%	25/1327	1.9%	1.45	0.81, 2.59	0.21
Open-angle glaucoma	39/1619	2.4%	32/1332	2.4%	0.99	0.62, 1.60	0.99
Angle-closure glaucoma	15/1619	0.9%	10/1332	0.8%	0.81	0.36, 1.80	0.60
Age-related macular degen.	97/1574	6.2%	64/1291	5.0%	0.79	0.57, 1.09	0.16
Early stage	95/1574	6.0%	59/1291	4.6%	0.75	0.53, 1.04	0.08
Late stage	2/1574	0.1%	5/1291	0.4%	3.07	0.60, 15.9	0.30

The denominators vary between various parameters, since these parameters could not be assessed in all subjects with a biochemical blood analysis.

95%CI: 95% confidence interval.

**Table 2:** Differences in Ocular Parameters in Subjects with and without Dyslipidemia in the Beijing Eye Study 2006 (Continuous variables).

	No Dyslipidemia Mean ± SD	Dyslipidemia (Mean ± SD)	Mean Difference	95% CI of the Difference	*P*- value
Best corrected visual acuity	1.08±3.23	0.93±0.25	0.14	−0.01, 0.30	0.07
Refractive error (Diopters)	−0.30±2.10	−0.31±2.46	0.01	−0.16, 0.18	0.91
Intraocular pressure (mm Hg)	15.36±2.90	15.93±3.00	−0.57	−0.78, −0.35	<0.001
Optic disc area (mm^2^)	2.55±0.50	2.63±0.51	−0.08	−0.12, −0.04	<0.001
Area of alpha zone area (mm^2^)	0.49±0.51	0.51±0.60	−0.02	−0.06, 0.02	0.39
Area of beta zone area (mm^2^)	0.29±0.95	0.44±1.45	−0.16	−0.25, −0.07	0.001
Neuroretinal rim area (mm^2^)	1.68±0.32	1.69±0.34	−0.01	−0.06, 0.04	0.69

CI: confidence interval; SD: standard deviation.

In a multivariate regression analysis adjusted for age, gender, area of habitation, body mass index, income, blood glucose concentration, diastolic blood pressure and smoking, dyslipidemia was significantly associated with higher intraocular pressure (β = 1.02; 95% CI: 1.01–1.02, *P*<0.001) and beta zone of parapapillary atrophy (β = 1.19; 95% CI: 1.02–1.39, *P* = 0.03) (Tables 3,4). In this multivariate analysis, dyslipidemia was not significantly associated with the prevalence of glaucoma (OR: 0.07, 95% CI: 0.0–0.15, *P* = 0.99), retinal vein occlusions (OR: 1.07; 95% CI: 0.29–3.99, *P* = 0.92), diabetic retinopathy (OR: 0.86; 95% CI: 0.57, 1.31, *P* = 0.49), and age-related macular degeneration (OR: 1.59, 95% CI: 0.70–3.64, *P* = 0.27), the presence of nuclear cataract (ß: 0.58, 95% CI: 0.28–1.19, *P* = 0.14), cortical cataract (ß: 1.03, 95% CI: 0.48–2.25, *P* = 0.93), and subcapsular cataract (ß: 0.80, 95% CI: 0.28–2.29, *P* = 0.67), and the presence of focal arteriolar narrowing of the retinal vessels (OR: 0.81, 95% CI: 0.45–1.45, *P* = 0.48), retinal arterio-venous nicking (OR: 0.89, 95% CI: 0.49–1.64, *P* = 0.71), and generalized retinal arteriolar narrowing (OR: 1.02, 95% CI: 0.65–1.61, *P* = 0.92) (Tables 3,4).

**Table 3:** Multivariate Regression Analysis of the Associations between the Presence of Dyslipidemia, Total Cholesterol, High-Density Lipoprotein Cholesterol, Low-Density Lipoprotein cholesterol, and Triglycerides with Major Ocular Diseases and Parameters in the Beijing Eye Study 2006.

Dyslipidemia				Triglycerides ( mmol/L)			Total Cholesterol (mmol/L)			High-Density Lipopr. (mmol/L)			Low-Density Lipopr. (mmol/L)		
	OR	95%CI	*P*-V.	OR	95%CI	*P*-V.	OR	95%CI	*P*-V.	OR	95%CI	*P*-Val.	OR	95%CI	*P*-V.
Ret. vein occlusion	1.07	0.29, 3.99	0.92	1.06	0.89, 1.27	0.51	1.14	0.84, 1.53	0.41	1.08	0.50, 2.33	0.85	1.23	0.93, 1.62	0.16
Open angle glaucoma	1.14	0.71, 1.84	0.99	0.99	0.81, 1.22	0.96	0.95	0.74, 1.22	0.70	0.84	0.40, 1.73	0.63	0.98	0.71, 1.35	0.91
Diab retinopathy	0.86	0.57, 1.31	0.49	0.92	0.77, 1.08	0.30	0.99	0.80, 1.24	0.99	1.96	0.92, 4.21	0.08	0.97	0.74, 1.29	0.86
Nuclear Cat.	0.58	0.28, 1.19	0.14	1.03	0.91, 1.16	0.62	0.97	0.82, 1.13	0.66	0.98	0.62, 1.53	0.93	0.99	0.81, 1.20	0.88
Cortical Cat.	1.03	0.48, 2.25	0.93	1.08	0.97, 1.20	0.19	0.94	0.81, 1.10	0.47	1.15	0.73, 1.79	0.55	0.93	0.77, 1.13	0.50
Post. subcaps. Cat.	0.80	0.28, 2.29	0.67	1.01	0.87, 1.18	0.87	0.96	0.78, 1.16	0.66	0.92	0.53, 1.59	0.77	0.91	0.71, 1.16	0.46
Age-related mac. degen.	1.59	0.70, 3.64	0.27	0.99	0.87, 1.13	0.93	0.96	0.81, 1.14	0.65	1.04	0.62, 1.73	0.90	0.87	0.71, 1.06	0.17
Focal retinal art. narrowing	0.81	0.45, 1.45	0.48	0.91	0.80, 1.03	0.15	0.95	0.82, 1.10	0.51	1.17	0.81, 1.69	0.41	0.91	0.77, 1.09	0.41
Retinal art- ven. nicking	0.89	0.49, 1.64	0.71	1.01	0.91, 1.10	0.83	1.05	0.91, 1.20	0.51	0.99	0.68, 1.46	0.97	1.00	0.85, 1.17	0.97
General. ret. art. narrowing	1.02	0.65, 1.61	0.92	1.04	0.97, 1.11	0.25	0.98	0.89, 1.08	0.70	0.63	0.46, 0.86	0.004	1.04	0.93, 1.17	0.49

(Categorical variables; logistic regression analysis) (OR: Odds ratio; 95%CI: 95% Confidence interval; *P*.-V.: *P*-Value).

**Table 4:** Multivariate Regression Analysis of the Associations between the Presence of Dyslipidemia, Total Cholesterol, High-Density Lipoprotein Cholesterol, Low-Density Lipoprotein cholesterol, and Triglycerides with Major Ocular Diseases and Parameters in the Beijing Eye Study 2006.

	Dyslipidemia			Triglycerides ( mmol/L)			Total Cholesterol (mmol/L)			High-Density Lipoprotein (mmol/L)			Low-Density Lipoprotein (mmol/L)		
	Beta Coeff.	95%CI	*P*-Val.	Beta Coeff.	95%CI	*P*-Val.	Beta Coeff.	95%CI	*P*-Val.	Beta Coeff.	95%CI	*P*-Val.	Beta	95%CI	P-Val.
Intraocular pressure (mm Hg)	1.02	1.01, 1.02	<0.001	0.19	0.10, 0.27	<0.001	0.26	0.15, 0.36	<0.001	0.21	0.06, 0.52	0.16	0.27	0.14, 0.39	<0.001
Alpha zone of para-papillary atrophy (mm^2^)	0.88	0.63, 1.23	0.47	0.01	0.01, 0.03	0.10	0.01	−0.01, 0.03	0.35	−0.03	−0.06, 0.03	0.32	0.01	−0.01, 0.04	0.33
Beta zone of para- papillary atrophy (mm^2^)	1.19	1.02, 1.39	0.03	0.00	−0.03, 0.03	0.84	0.03	−0.01, 0.07	0.10	0.05	−0.06, 0.16	0.40	−0.03	−0.08, 0.02	0.31

(Continuous variables; linear regression analysis) (95%CI: 95% Confidence interval; *P*.-V.: *P*-Value).

Each model was adjusted for factors significantly associated with each of the ocular parameters/diseases in bivariate analysis.

95%CI: 95% confidence interval of the odds ratio or of Beta coefficient.

If the multivariate statistical analysis included dyslipidemia as dependent parameter and age, diastolic blood pressure and intraocular pressure as independent variables, dyslipidemia remained to be significantly associated with higher intraocular pressure (*P*<0.001) in addition to higher age (*P*<0.001) and higher diastolic blood pressure (*P*<0.001). If the whole study population was stratified into age groups of each 10 years each, the correlation between dyslipidemia and higher intraocular pressure (adjusted for diastolic blood pressure) remained to be statistically significant in the age group from 45–54 years (*P* = 0.02; ß = 1.05; 95%CI: 1.01, 1.09) and in the age group from 55–64 years (*P* = 0.01; ß = 1.06; 95%CI: 1.01, 1.1). In the remaining age groups, the association was not statistically significant, potentially due to the decreasing number of participants per group. If the whole study population was divided into two age groups with the cut-off at 60 years, the association between dyslipidemia and intraocular pressure was significant in the younger subgroup (*P*<0.001) and in the elder subgroup (*P* = 0.04). If the whole study population was stratified into participants without or with arterial hypertension, the relationship between dyslipidemia and intraocular pressure was significant in the non-arterial hypertensive group. If the whole study population was stratified into participants without or with diabetes mellitus, the relationship between dyslipidemia and intraocular pressure was significant in the non-diabetic group.

If the statistical analysis defined dyslipidemia as abnormal lipid concentrations (not including the history of abnormal lipid measurements), similar results were obtained as if the definition of dyslipidemia included the history of it. In univariate analysis, we found significant associations for the relationships between dyslipidemia (without taking into account history of dyslipidemia) with intraocular pressure (*P*<0.001; correlation coefficient: 0.10) and with beta zone of parapapillary atrophy (*P* = 0.001; correlation coefficient: 0.06). For all other ocular diseases, the relationship was not statistically significant. In a multivariate regression analysis adjusted for age, gender, area of habitation, body mass index, income, blood glucose concentration, diastolic blood pressure and smoking, the prevalence of dislipidemia (not including the history of abnormal lipid measurements) was significantly associated with higher intraocular pressure (β = 1.06; 95% CI: 1.03–1.09, *P*<0.001) and beta zone of parapapillary atrophy (β = 1.12; 95% CI: 1.05–1.20, *P* = 0.002).

## Discussion

Our study was performed in search of associations between dyslipidemia and ocular parameters and diseases. It revealed a correlation of dyslipidemia with intraocular pressure and with beta zone of parapapillary atrophy.

The finding of a relationship between dyslipidemia and increased intraocular pressure has not been reported yet and therefore is in need of further confirmation. Interestingly, despite the association between dyslipidemia and intraocular pressure, dyslipidemia was not associated with the presence of glaucomatous optic neuropathy. In previous studies, similar findings were reported for the association between increased blood pressure and intraocular pressure: although arterial hypertension was associated with increased intraocular pressure, it was not associated with glaucoma [23]–[26]. The finding of a relationship between dyslipidemia and parapapillary atrophy (beta zone) has neither been reported yet. This observation has to be considered to be relatively vague, until confirmed in future investigations. Interestingly, although beta zone of parapapillary atrophy was related to glaucomatous optic neuropathy [27], [28], dyslipidemia was not associated with glaucoma.

Dyslipidemia was not significantly associated with the prevalence of diabetic retinopathy after controlling for systemic parameters of age, gender, body mass index, blood glucose concentration, diastolic blood pressure and smoking. The finding appears to disagree with results of previous population-based studies and of clinical investigations which showed a disappearance of retinal hard exudates in patients with diabetic retinopathy after lowering of systemic blood lipid concentrations [29], [30]. One of the reasons for the discrepancy between the studies may be differences in lifestyle and food between adult Chinese participating in our study and adult Caucasians taking part in the previous population-based studies. Another reason may be that the number of subjects with hard exudates as part of diabetic retinopathy in our study was too small for a statistical analysis. The missing of a clearly significant association between dyslipidemia and the overall presence of diabetic retinopathy in our study may suggest that factors such as blood glucose concentration, blood pressure, region of habitation and gender were more important than the additional factor of dyslipidemia for the development of diabetic fundus changes.

The result of our study that dyslipidemia was not associated with the presence of age-related macular degeneration agrees with data from the Complications of Age-Related Macular Degeneration Prevention Trial that showed that use of the cholesterol-lowering medications among patients with bilateral macular drusen was not associated with a higher or lower risk of progressing to advanced disease [31].

The observation that retinal vascular abnormalities (i.e. focal arteriolar narrowing, retinal arterio-venous nicking and generalized retinal arteriolar narrowing) were not significantly associated with the presence of dyslipidemia after controlling for the systemic factors of blood glucose concentration, diastolic blood pressure and smoking, confirmed a previous study by Leung and colleagues in which no consistent pattern of an association between serum total cholesterol or low-density lipoprotein cholesterol and any retinal microvascular signs was detected [32].

Potential limitations of our study should be mentioned. First, a major concern regarding any epidemiologic study is non-participation. The Beijing Eye Study 2006 had a reasonable response rate of about 73% (3251 of the 4439 subjects invited for the follow-up survey; or 61% of the group originally eligible in 2001). However, differences between participants and non-participants could have led to a selection artifact. Second, the number of subjects with some diseases, e.g., late age-related macular degeneration, was small so that the statistical power was not sufficient to exclude a weak association between the disease in question and dyslipidemia. However, we had adequate statistical power to determine associations of dyslipidemia to any age-related macular degeneration, glaucoma and age-related cataract. Third, a further limitation of the study design was that socioeconomic parameters, such as income, were assessed based on an interview, without re-checking in a more scientific manner. Fourth, the prevalence of dyslipidemia was 56%, a value higher than what might have been expected. Interestingly, however, the results of our study agree with previous populations-based studies from China. To cite an example, if the results of our study were compared with the findings from the InterAsia Collaborative Group Study [33], the mean levels of total cholesterol, HDL cholesterol, LDL cholesterol, and triyglcerides did not vary markedly between both studies. The prevalence of dyslipidemia and its associations found in our study also agreed with other studies from China such as the PRC-USA Collaborative Study in Cardiovascular and Cardiopulmonary Epidemiology and other investigations [34]–[36]. Fifth, our study was a cross-sectional investigation which primarily did not allow statements about longitudinal relationships. Sixth, it has to be considered, that a relatively high number of statistical comparisons were performed, so that a Bonferroni correction should be performed. It would reveal that the association between dyslipidemia and intraocular pressure continued to be significant, while the association between dyslipidemia and beta zone of parapapillary atrophy would no longer be statistically significant. Strengths of our study are that dyslipidemia was adjusted for major systemic parameters including socioeconomic factors so that it may have been unlikely that inter-relationships between dyslipidemia and non-ocular parameters may have led to a confounding effect; that it was the first investigation on an association between dyslipidemia and major eye diseases in China; the relatively large study sample; and the application of internationally accepted definitions for ophthalmic diseases, such as the glaucoma definition by the International Society of Geographic and Epidemiological Ophthalmology ISGEO [37].

In conclusion, after adjusting for systemic and socioeconomic parameters, dyslipidemia in Chinese individuals was associated with increased intraocular pressure, diabetic retinopathy and parapapillary atrophy (beta zone). However, dyslipidemia was not associated significantly with the prevalence of glaucoma, retinal vein occlusions, and age-related macular degeneration, any type of age-related cataract, and the presence of retinal vascular abnormalities.

## Methods

### Ethics Statement

The Medical Ethics Committee of Beijing Tongren Hospital approved the study protocol and all participants gave informed consent according to the Declaration of Helsinki.

The Beijing Eye Study was a population-based study conducted in rural and urban regions of Greater Beijing. It has been described in detail recently [38]–[40]. At the time of the first survey in the year 2001, out of 5324 eligible subjects with an age of 40+ years, 4439 individuals participated (response rate: 83.4%). The current study population was examined in 2006, when participants of the survey of 2001 were invited for re-examination. Of the 4439 subjects who participated at baseline in 2001, 3251 subjects returned for the follow-up examination in 2006 (response rate: 73.2%).

All examinations were carried out in school houses or in common houses of the seven communities included. Trained research technicians asked the study participants questions from a standard questionnaire providing information on demographic variables such as age, gender, level of education, occupation, family income; on smoking and alcohol drinking habits; and on known diagnosis and current treatment of arterial hypertension and dyslipidemia. Body weight and height and the arterial blood pressure were measured. At the start of the examinations in the morning, fasting blood samples were taken and the fresh blood samples were biochemically analyzed within at maximum four hours. In the fasting blood samples, the serum concentrations of total cholesterol, triglycerides, high-density lipoprotein-cholesterol (HDL-C) and low-density lipoprotein-cholesterol (LDL-C) were measured using the enzymatic method with a Hitachi 7600 auto-analyzer (Hitachi, Tokyo, Japan). Reagents of the same batch were used. Dyslipidemia was defined as any of hypercholesterolemia (total cholesterol concentration ≥5.72 mmol/L (220 mg/dL)) or hypertriglyceridemia (triglyceride concentration ≥1.70 mmol/L (150 mg/dL)) or low high-density lipoprotein-cholesterol (HDL-C concentration ≤0.91 mmol/L (35 mg/dL)), or as treatment of dyslipidemia [33]. Treatment of dyslipidemia was defined as the use of a pharmacological treatment to lower blood lipids during the previous 2 weeks.

A detailed ophthalmic examination was performed including refractometry, frequency doubling perimetry, slit lamp assisted optical coherence tomography of the anterior chamber morphology (Heidelberg Engineering, Heidelberg, Germany), ophthalmoscopy, and photography of the cornea, lens and fundus. Intraocular pressure was measured by a non-contact pneumotonometer (CT-60 computerized tonometer, Topcon Ltd., Tokyo, Japan). The anterior chamber depth was assessed using the method described by van Herick et al. [41]. Examining the lens photographs, nuclear cataract was graded in 6 grades based on the classification scheme of the Age-Related Eye Disease Study [42]. Nuclear cataract was defined as a grade of nuclear cataract of “4” or higher. The degree of cortical lens opacification and posterior subcapsular lens opacification was graded using photographs taken by retro-illumination with the Neitz CT-R camera (Neitz Instruments Co., Tokyo, Japan). For the statistical analysis, cortical cataract and posterior subcapsular cataract were defined as any presence of cortical cataract or posterior subcapsular cataract, respectively. The occurrence of retinal vein occlusions was assessed using fundus photographs [43]. Retinopathy lesions were examined using criteria of the Early Treatment of Diabetic Retinopathy Study (ETDRS) [44], [45].

Monoscopic photographs (on film) of the macula and optic disc were taken using a fundus camera (Type CR6-45NM, Canon Inc. U.S.A.). The optic disc photographs were digitized and the width of the neuroretinal rim and the diameters and area of the optic cup and optic disc were measured in the vertical meridian of the optic disc. Additionally, we measured the area of beta zone of parapapillary atrophy [46]. The vertical cup / disc diameter ratio (VCDR) was calculated. Glaucoma was defined according to the criteria of the International Society of Geographic and Epidemiological Ophthalmology ISGEO [37]. The differentiation between open-angle glaucoma (OAG) and angle-closure glaucoma (ACG) was performed according to Goldmann gonioscopy, which was carried out for all glaucoma subjects and glaucoma suspects, and according to the images of the anterior segment optical coherence tomography. For the evaluation of retinal vascular abnormalities, focal narrowing of arterioles, generalized narrowing of arterioles, and arteriovenous crossing abnormalities (arteriovenous nicking) were assessed using the protocol of the Atherosclerosis Risk in Communities (ARIC) study [47], [48]. For the assessment of age-related macular degeneration, the Wisconsin Age-Related Maculopathy Grading system was applied [49].

Statistical analysis was performed using SPSS (SPSS for Windows, version 17.0, SPSS, Chicago, IL). Continuous data were presented as mean ± standard deviation. Student t tests were performed to compare mean values. In the first part of the analysis, the presence of dyslipidemia was the solely (categorical) exposure parameter (Tables 1, 2, 3, 4). In the second part of the analysis, the blood concentrations of total cholesterol, high-density lipoprotein cholesterol, low-density lipoprotein cholesterol and triglycerides served separately as continuous exposure parameters (Tables 3,4). The outcome parameters were either categorical variables (such as the presence of glaucoma) or continuous variables (such as intraocular pressure. Simple / bivariate logistic or linear regression models were used to investigate the associations of the presence of dyslipidemia with the continuous (e.g., intraocular pressure) or categorical outcomes (e.g., diabetic retinopathy). As confounding variables we choose parameters which either as systemic parameters could be expected to be associated with dyslipidemia, such as age and gender, or ocular parameters which could be expected to be associated either with dyslipidemia or with other ocular parameters which could be associated with dyslipidemia. In a second step of the statistical analysis, multivariate models were calculated which included the systemic and demographic variables of age, gender, area of habitation, body mass index, income, blood glucose concentration, diastolic blood pressure and smoking. For continuous variables, beta coefficients (β) were reported; for categorical variables, odds ratios (OR) were presented, and 95% confidence intervals (CI) were described. All *P*-values were 2-sided and were considered statistically significant when the values were less than 0.05.
